# ICTIS: A Novel Scoring System to Assess the Inclusivity of Advanced NSCLC Immunotherapy Trials

**DOI:** 10.1016/j.jtocrr.2025.100878

**Published:** 2025-07-12

**Authors:** Kira Nguyen, Ashley Wei, Srinivas Govindan, Eziafa Oduah, Nagashree Seetharamu, Wint Yan Aung

**Affiliations:** a Zuckerberg Cancer Center, Northwell Health, Donald and Barbara Zucker School of Medicine at Hofstra/Northwell, 450 Lakeville Rd, Lake Success, New York; b Department of Medicine, Donald and Barbara Zucker School of Medicine at Hofstra/Northwell, 300 Community Drive, Manhasset, New York; c Division of Medical Oncology, Duke Cancer Institute, Duke University School of Medicine, Durham, North Carolina

**Keywords:** Clinical trial inclusivity, Eligibility criteria, Immunotherapy, Non–small cell lung cancer

## Abstract

**Introduction:**

Immunotherapy has revolutionized the treatment of NSCLC. However, trials that led to approval of these agents and ongoing trials often include overly included overly restrictive exclusion criteria, limiting access for a significant proportion of patients. We propose the immunotherapy clinical trial inclusivity score (ICTIS), a scoring system to evaluate trial eligibility criteria for inclusivity.

**Methods:**

ICTIS was developed using national guidelines and validated with a Cohen’s Kappa statistics of 0.807. Eligibility criteria for advanced NSCLC immunotherapy trials on ClinicalTrials.gov were scored using a binary scale (0 = exclusive, 1 = inclusive), with higher summed scores indicating higher inclusivity. Mean ICTIS scores were compared across lines of treatment, start date, and trial phase.

**Results:**

The mean ICTIS score among 142 trials was 12.7 (SD 4), with 28 trials (19.7%) rated as excellent and 34 trials (23.9%) rated poor. The most restrictive criteria were Eastern Cooperative Oncology Group performance status (78.8%), organ function criteria of bilirubin (76.1%), and absolute neutrophil count (65.5%). First-line trials were significantly more exclusive to patients with pneumonitis history, with 64% exclusion versus 45.5% in second-line (χ^2^ = 4.917, *p* = 0.027). The platelet count requirement was more stringent in monotherapy trials. Inclusion of treated leptomeningeal disease improved over time (χ^2^ = 7.99, *p* = 0.018), but eligibility criteria remained consistent across different time periods, lines of treatment, and trial phases.

**Conclusions:**

Despite the release of national guidelines, immunotherapy trials have overall retained restrictive eligibility criteria. ICTIS provides a standardized framework for evaluating inclusivity and can assist in designing immunotherapy studies to be more inclusive.

## Introduction

Lung cancer is the leading cause of cancer death, accounting for 124,730 deaths (20.2% of all cancer deaths) in the United States in 2025. More than 90% of patients with lung cancer are diagnosed at a late, advanced stage in the United States.^1^ For advanced or metastatic NSCLC, the reported 5-year survival rate is only 9.7%, emphasizing the need for more effective treatments.^2^ The treatment landscape for advanced NSCLC has evolved considerably with the integration of chemotherapy, targeted therapy, immunotherapy, or a combination of the above.^3^^,^^4^ Immune checkpoint inhibitors (ICIs) form the most important class of immunotherapy agents and significantly improved overall survival for many patients with NSCLC without actionable mutations.^3^, ^4^, ^5^

Many clinical trials have reported clear benefits of ICIs in advanced NSCLC. For example, Hellmann et al.^5^ reported that 42.6% of patients with advanced NSCLC remained progression-free at 1 year with ICI agents nivolumab and ipilimumab, compared with 13.2% of patients receiving chemotherapy. Similarly, the phase 3 KEYNOTE-024 (Pembrolizumab versus Chemotherapy for PD-L1–Positive Non–Small-Cell Lung Cancer) trial reported improved overall survival (median 30 versus 14.2 mo) and response rate (44.8% versus 27.8%) in patients treated with pembrolizumab monotherapy versus platinum-based chemotherapy. This KEYNOTE-024 study produced an unprecedented 31.9% 5-year survival rate, proving the groundbreaking potential of ICIs.^6^ In the phase 3 KEYNOTE-189 (Pembrolizumab plus Chemotherapy in Metastatic Non–Small-Cell Lung Cancer) trial, pembrolizumab combined with chemotherapy outperformed chemotherapy plus placebo with significantly improved overall survival (median 22.0 versus 10.7 mo) and progression-free survival (9.0 versus 4.9 mo).^7^

These immunotherapy trials contributed to a paradigm shift in the treatment of NSCLC and improved outcomes.^8^ Despite these advancements, clinical trials continue to be the optimal treatment option for patients with advanced NSCLC. Design of clinical trials requires well-defined eligibility criteria to minimize confounding variables, enhance patient safety, and increase internal validity.^9^ A key challenge with immunotherapy clinical trials, including those that have led to approvals, is overly strict eligibility criteria. Many of these criteria were originally established for cytotoxic chemotherapy studies and have been carried over to immunotherapy trials despite the unique characteristics of immune-based therapies.^10^^,^^11^ As a result, these stringent requirements may unnecessarily limit patient enrollment and decrease the external validity of these studies.^9^^,^^11^

When trial populations are overly restrictive and do not reflect real-world patient populations, it hinders accurate assessment of a drug’s efficacy and safety in the populations that it aims to treat.^12^ For example, Cramer-van der Welle et al.^13^ noted that the median overall survival for patients with first-line advanced NSCLC treated with pembrolizumab in real-world settings was significantly lower (15.8 mo) compared with the clinical trial setting (30.0 mo). Similarly, in patients with second-line advanced NSCLC treated with nivolumab, the real-world median overall survival (8.2 mo) was lower than the trial-reported median (12.2 mo), though this difference was not statistically significant. A study by Gan et al.^14^ retrospectively analyzed outcomes between trial-eligible and trial-ineligible patients with advanced NSCLC, renal cell carcinoma, and melanoma. On the basis of common exclusion criteria in immunotherapy trials, 68% of patients were deemed trial-eligible and 32% as ineligible. For NSCLC, trial-ineligible patients had significantly lower overall survival (5.3 mo versus 20.4 mo), shorter treatment duration (2.1 mo versus 5.9 mo), and shorter time to next treatment (4.7 mo versus 10.3 mo) compared with their trial-eligible counterparts. These studies highlight the significant discrepancy between outcomes observed in clinical trials and in real-world clinical settings.

Leading experts and national organizations have advocated fundamental changes to expand eligibility criteria in clinical trials, aiming to safely include a more representative patient population and improve access to immunotherapy. Groups such as the American Society of Clinical Oncology (ASCO), Friends of Cancer Research (FOCR), the U.S. Food and Drug Administration, and LUNGevity—comprising clinicians, clinical pharmacologists, patient advocates, and representatives from regulatory and industry sectors—have proposed national recommendations to bridge the efficacy gap, enhance participant diversity, and improve trial participation.^10^^,^^15^

An example of restrictive criteria is limiting trial patients to those with a high-performance status using the Eastern Cooperative Oncology Group (ECOG) performance status of 0 to 1. This criterion disproportionately excludes older adults, as patients with ECOG 2 have a median age of 72 years, compared with 65 years for those with ECOG 0 to 1.^16^ However, expert opinions in a review by Gridelli et al.^17^ noted that the exclusion of ECOG 2 patients was originally developed for chemotherapy, and ECOG 2 status does not predict adverse events in immunotherapy. ASCO and FOCR’s 2021 guidelines now recommend including ECOG 2 patients in trials unless scientific evidence suggests otherwise.^15^ In addition, immunotherapy trials often exclude patients with human immunodeficiency virus (HIV) because of concerns about immune-related adverse events. Uldrick et al.^18^ reported that patients with advanced-stage HIV on antiretroviral therapy experienced adverse event rates comparable to HIV-negative patients. Similarly, Cook et al.^19^ reported that 93% of patients maintained suppressed HIV viral loads during immunotherapy, supporting the inclusion of HIV-positive patients in these trials. Notably, by expanding the three criteria per ASCO-FOCR’s 2017 guidelines ([1] previous or concurrent malignancies, [2] brain metastases, and [3] poor renal function), potential trial eligibility for patients with advanced NSCLC increased from 52% (5495 patients) to 98% (10,346 patients).^20^

Despite these guidelines, there is no standard metric to evaluate the extent to which NSCLC immunotherapy clinical trials have followed the recommendations. In this study, we aimed to address this gap by (1) identifying the most common restrictive eligibility criteria, and (2) assessing the inclusivity of current NSCLC immunotherapy trials using a scoring system. The immunotherapy clinical trial inclusivity scale (ICTIS) was developed by integrating existing national recommendations to provide a tool for evaluating inclusivity and quality of an immunotherapy trial design.

## Materials and Methods

### Development of ICTIS

Recommendations from ASCO, Friends of Cancer Research, FDA, and LUNGevity informed the development of ICTIS.^10^^,^^11^^,^^15^^,^^20^ Common eligibility criteria were identified from ClinicalTrials.gov and from a literature review of immunotherapy and advanced lung cancer-specific criteria. These sources informed the development of the ICTIS scoring system.

ICTIS is a summative scale consisting of 22 criteria with a maximum possible score of 22. A version of ICTIS is presented in Table 1. To calculate a trial’s ICTIS score, the scorer assigns either one point or zero point to each of the 22 criteria. A higher score reflects greater inclusivity in the trial’s eligibility criteria.

**Table 1:** Abbreviated Immunotherapy Clinical Trial Inclusivity Scale (ICTIS)

Criteria	1 Point Given: Inclusive	0 Points Given: Exclusive
Demographic and general		
ECOG Performance Status	Performance status 2 or above maximum	Performance status 0 or 1 maximum
Psychiatric	No psychiatric or social criteria are listed in the protocol (not mentioned)	Psychiatric of social criteria are mentioned in the protocol
Life Expectancy	No life expectancy criteria are listed in the protocol (not mentioned)	Life expectancy criteria are mentioned in the protocol
Age upper limit	Upper limit ≥85 years old OR not mentioned	Upper limit <85 years old
Organ Function		
Platelets, immunotherapy only	The minimum requirement for platelets is less than or equal to 75,000 cells/μL (patients with 75,000 platelets/μL would be included in the study) OR not mentioned	The minimum requirement for platelets is greater than 75,000 cells/μL (patients with 75,000 platelets/μL would be excluded from the study)
Platelets, chemo, radiation, or targeted combination therapy	The minimum requirement for platelets is less than or equal to 100,000 cells/μL (patients with 100,000 platelets/μL would be included in the study) OR not mentioned	The minimum requirement for platelets is greater than 100,000 cells/μL (patients with 100,000 platelets/μL would be excluded from the study) OR not mentioned
Hemoglobin	The minimum requirement for hemoglobin is less than or equal to ≤8 g/dL (patients with 8 g/dL would be included in the study) OR not mentioned	The minimum requirement for hemoglobin is greater than ≤8g/dLcells/μL (patients with 8 g/dL would be excluded from the study)
AST or ALT	The maximum AST or ALT inclusion is greater than or equal to 3 x ULN (patients with 3 x ULN AST or ALT would be included in the study) OR not mentioned	The maximum AST or ALT inclusion is less than or equal to 3 x ULN (patients with 3 x ULN AST or ALT would be excluded from the study)
AST or ALT, liver metastases	The maximum AST or ALT inclusion in patients with liver metastases is greater than or equal to 5 x ULN (patients with liver metastases and 5x ULN AST or ALT would be included in the study) OR not mentioned	The maximum AST or ALT inclusion in patients with liver metastases is less than or equal to 5 x ULN (patients with liver metastases and 5x ULN AST or ALT would be included in the study) OR not mentioned patients with liver metastases are not mentioned when AST or ALT is mentioned and has a less than maximum requirement of more than 5x ULN
Creatinine	The minimum creatinine clearance requirement is less than or equal to 30 mL/min (patients with 30mL/min creatinine clearance would be included in the study) OR not mentioned	The minimum creatinine clearance requirement is greater than or equal to 30 mL/min (patients 30mL/min creatinine clearance would be excluded from the study) OR not mentioned
Bilirubin	Not mentioned, no bilirubin requirement	Any bilirubin requirement
Absolute Neutrophil Count	The minimum requirement for absolute neutrophil count is less than or equal to ≤ 1000 cells/μL (patients with 1000 cells/μL would be included in the study) OR not mentioned	The minimum requirement for absolute neutrophil count is greater than ≤ 1000 cells/μL (patients with 1000 cells/μL would be excluded from the study)
Cardiac	Validated clinical classification system used (e.g., New York Heart Association Functional Classification) OR low-risk cardiac events occurring ≤ 3 months excluded OR not mentioned	Vague or unclear exclusion not using validated clinical classification system (e.g., significant cardiovascular event) OR low-risk cardiac events occurring > 3+ months excluded
Treatment		
Washout Period	For chemo or targeted combination therapies: Washout period ≤21 days OR not mentioned	For chemo or targeted combination therapies: Washout period >21 days OR nonspecific washout periods (eg, 5 half lives)
	For radiation combination therapies or immunotherapy monotherapy: Washout period not mentioned	For radiation combination therapies or immunotherapy monotherapy: Any washout period restrictions
Comorbidities		
∗Pneumonitis	Infections (pneumonia) that have resolved spontaneously or with antibiotics included AND radiation pneumonitis that has subsided and does not require ongoing corticosteroid treatment included OR pneumonitis not mentioned	Resolved pneumonia, either spontaneously or with antibiotics excluded OR radiation pneumonitis that has subsided and doesn’t require ongoing corticosteroid excluded
Previous or Concurrent Malignancy	Only malignancies with a natural history or treatment has the potential to interfere with the safety or efficacy assessment of the investigational regimen are excluded, and included previous or concurrent malignancies should be listed out OR not mentioned	All previous malignancies are excluded OR vague criteria that does not specify which malignancies are excluded
CNS Metastases	Patients with treated or asymptomatic brain metastases are included OR brain metastases not mentioned	All patients with brain metastases excluded
Leptomeningeal	Treated (or doesn’t require treatment during trial) leptomeningeal disease included OR not mentioned	Treated (or doesn’t require treatment during trial) leptomeningeal disease excluded
Infection	Includes well-controlled infections OR infections not mentioned	All infections excluded OR a severe or active infection is not defined (either through examples)
HIV	All HIV included OR HIV included on effective antiretroviral therapy with undetectable viral load within 6 months OR HIV not mentioned	All HIV excluded, history of HIV excluded
Hepatitis B	Treated hepatitis B included OR undetectable HBV viral load included OR only positive PCR test excluded OR not mentioned	Excluded any positive test
Hepatitis C	Treated Hepatitis C included OR undetectable HCV viral load included OR only positive PCR test excluded OR not mentioned	Excludes any positive antibody test for HCV
Autoimmune Disease	Includes all autoimmune patients OR only excludes patients on immunosuppressants (≤1 year of use) OR only excluded active autoimmune diseases OR autoimmune diseases not mentioned	Patients excluded even without immunosuppressants OR patients excluded for immunosuppressant use >1 year before the trial begins

Scoring system points: OR means either of the criteria can be fulfilled to be awarded the point, as long as no exclusive criteria are mentioned. AND means both criteria must be fulfilled to be awarded the point. Zero points are awarded if any exclusive criteria are mentioned, regardless of whether inclusive criteria are mentioned for each category. Zero points are awarded if any criteria are vague (e.g., “adequate” organ function, no laboratory values listed, no explanation of what “severe” means).

ALT, alanine aminotransferase; AST, aspartate aminotransferase; chemo, chemotherapy; ECOG, Eastern Cooperative Oncology Group; HCV, hepatitis C virus; HIV, human immunodeficiency virus; PCR, polymerase chain reaction; ULN, upper limit of normal.

To assess the inclusiveness of clinical trials on the basis of ICTIS, we queried the ClinicalTrials.gov database for advanced NSCLC trials in October 2022, which identified 343 studies. The eligibility criteria of each trial were reviewed to include immunotherapy trials and used to score trials using the ICTIS scoring system. No patient data was accessed for this study.

### Validation of ICTIS

To validate ICTIS, two investigators (AW and KN) independently scored 50 trials. Interrater reliability was assessed using the Cohen’s Kappa coefficient. Scoring criteria were refined through consensus among investigators. Each criterion follows a binary system with one point awarded for an inclusive criterion and zero points for an exclusive criterion. Trials were categorized as poor, good, and excellent on the basis of the distribution of scores.

### Application of ICTIS

Advanced NSCLC immunotherapy clinical trials extracted from ClinicalTrials.gov were scored by participating investigators using the ICTIS scoring system. For each ICTIS criterion, we calculated the proportion of trials that were inclusive and exclusive. Chi-square tests were conducted to compare specific ICTIS criteria across subgroups, including line of treatment, start date, treatment type, and trial phase. Summary statistics, including mean, median, interquartile range (IQR), and SD, were calculated for ICTIS scores. Mean ICTIS scores were compared across subgroups to determine differences in inclusivity of eligibility criteria using *t* tests and analysis of variance.

### ClinicalTrials.gov Concordance With Full Criteria

The eligibility criteria from 17 publicly available full protocols were compared with those listed on ClinicalTrials.gov to assess the accuracy of the information reported on the website. The number of different criteria and the impact of the differences on ICTIS were measured. A two-tailed paired *t* test was run to compare the ClinicalTrials.gov ICTIS score with those of the full protocols.

## Results

### Overview of Extracted Recruiting NSCLC Immunotherapy Trials

**Figure 1:**
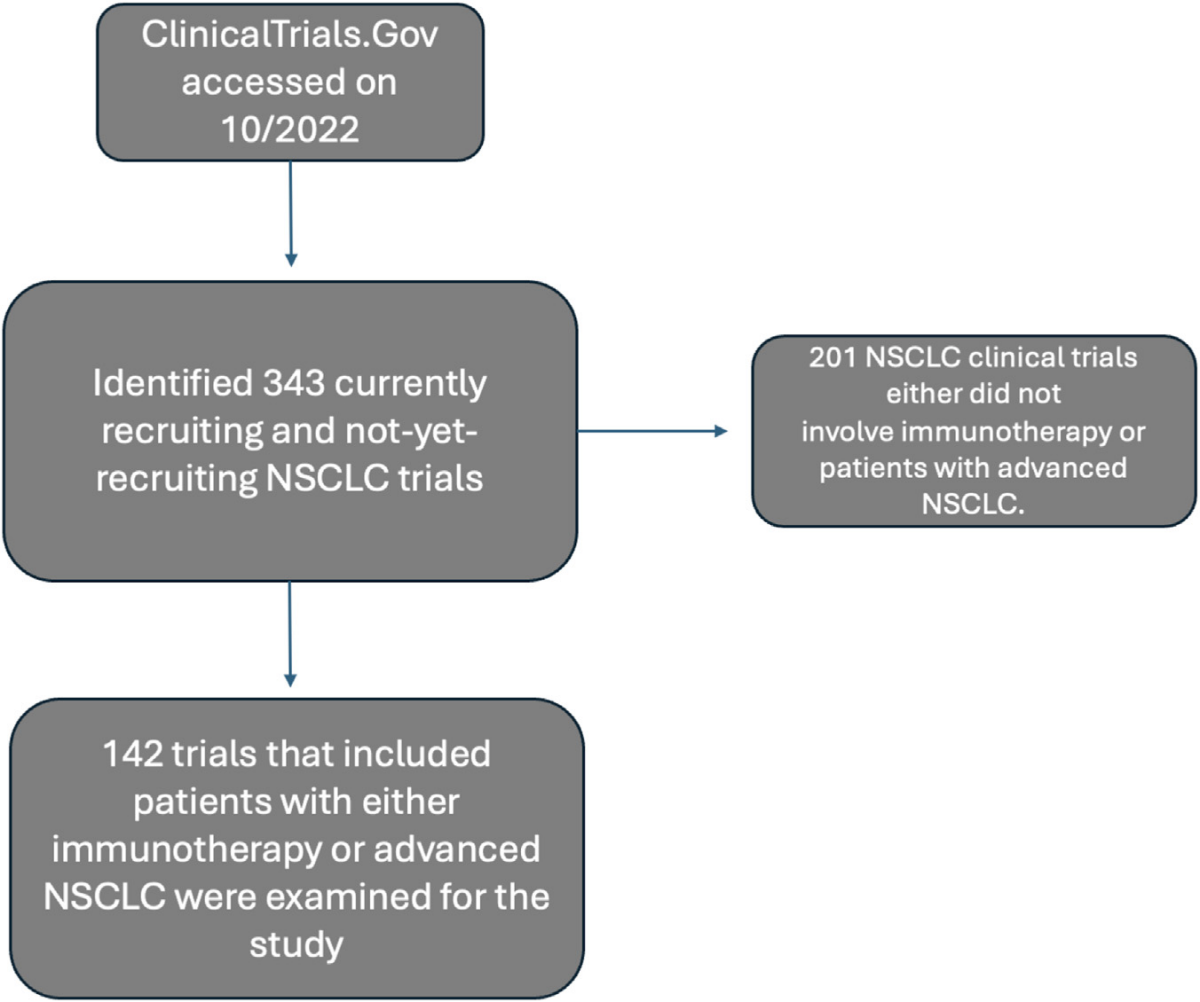
Flow diagram of advanced NSCLC immunotherapy trials accessed from ClinicalTrials.gov.

Out of the 343 currently recruiting and not-yet-recruiting NSCLC trials accessed from ClinicalTrials.gov, 142 trials focusing on advanced NSCLC immunotherapy clinical trials were selected (Fig. 1). The remaining 201 NSCLC clinical trials did not involve immunotherapy or patients with advanced NSCLC. These 142 trials were evaluated to evaluate the current state of eligibility criteria among immunotherapy trials in advanced NSCLC. Table 2 details the characteristics of these trials. Among these trials, 36.7% investigated chemoimmunotherapy, 29.1% immunotherapy alone, 24% in combination with targeted therapy, and 11% in combination with radiation. Notably, most of these trials were in a second-line setting (63.5%) compared with a first-line setting (36.4%). There were 49.2% of trials which were initiated between 2021 and 2023, and 46.4% between 2017 and 2020, after the release of major guidelines from ASCO and FOCR. These time frames were chosen in reference to the release of the first ASCO and FOCR guidelines in 2017 and their subsequent update in 2021.^11^^,^^15^

**Table 2:** Frequency of recruiting and not-yet-recruiting immunotherapy clinical trials for unresectable NSCLC. Some trials may fulfill more than one criterion (N = 142)

Sponsor Type	n (%)
Federal	107 (66.8)
Investigator	29 (18.1)
Industry	24 (15.0)
**Treatment Type**	
Immunotherapy Alone	46 (29.1)
Chemotherapy Combination	58 (36.7)
Targeted Combination Therapy	38 (24.0)
Radiation Combination Therapy	11 (6.9)
Other Combination Therapy	5 (3.1)
**Line of Trial**	
First-Line	58 (36.4)
Second-Line	101 (63.5)
**Start Year**	
Before 2017	6 (4.2)
2017 - 2020	66 (46.4)
2021 - 2023	70 (49.2)
**Phase**	
Phase 1	53 (36.0)
Phase 2	68 (46.2)
Phase 3	26 (17.6)
**Stage of Disease**	
Stage IV only	60 (42.2)
Stage III & IV	82 (57.7)

### Validation of ICTIS

ICTIS is a summative scale consisting of 22 criteria with a maximum possible score of 22 (Table 1). A total of 50 trials were independently scored by two investigators, with Cohen’s Kappa Coefficient was 0.807, suggesting high interrater agreement and reliability. To calculate a trial’s ICTIS score, the scorer assigns either one point or zero points to each of the 22 criteria. A higher score reflects greater inclusivity in the trial’s eligibility criteria.

### ICTIS Scores Across NSCLC Immunotherapy Clinical Trials

**Figure 2:**
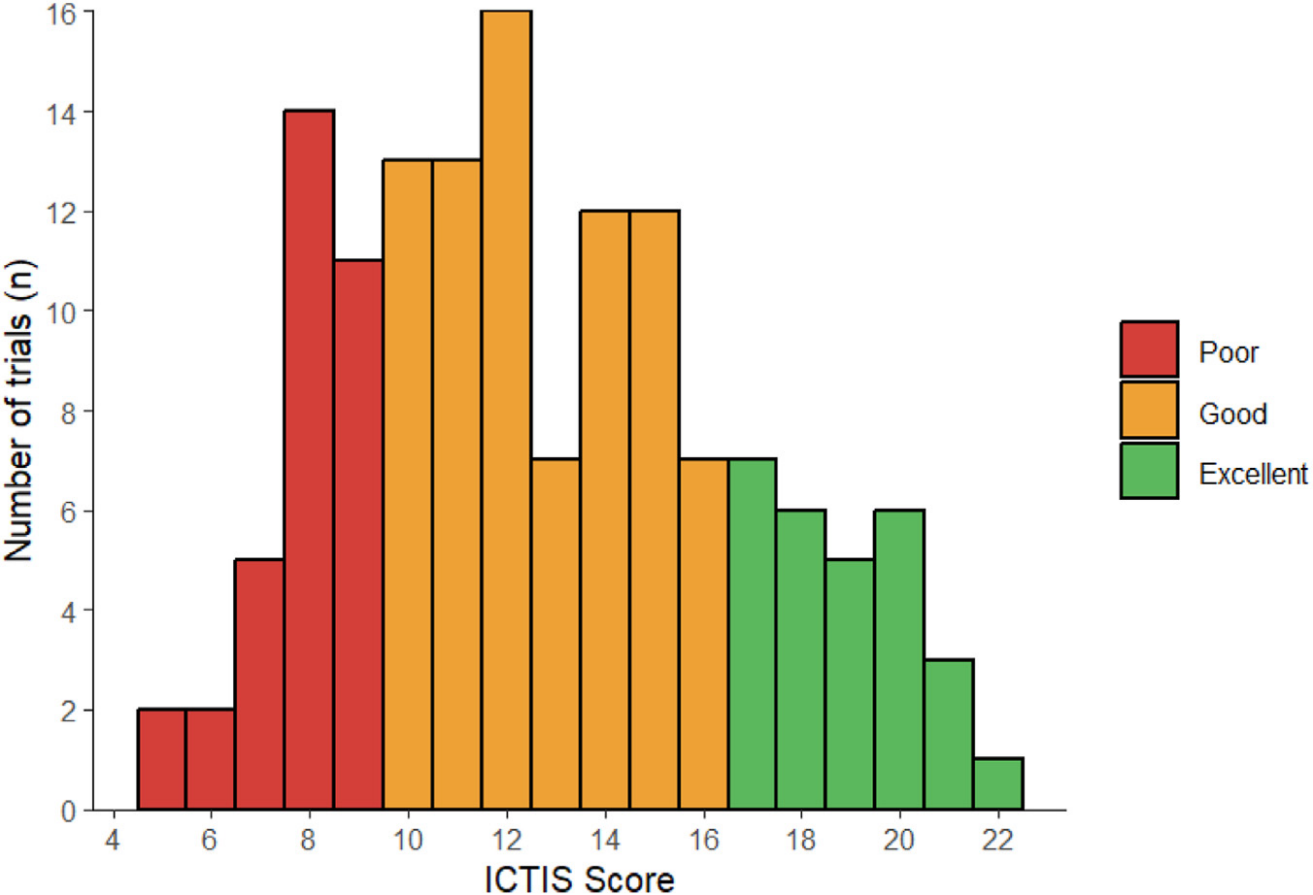
Frequency of ICTIS scores among the 142 NSCLC immunotherapy clinical trials. ICTIS, immunotherapy clinical trial inclusivity scale.

Figure 2 illustrates the distribution of ICTIS scores across 142 NSCLC immunotherapy clinical trials, which were accessed from ClinicalTrials.gov. The ICTIS scores are categorized into three groups: poor (0–9), good (10–16), and excellent (17–22). The mean ICTIS score across all 142 trials was 12.7 (SD = 4). The median was 12 with an IQR of 10 to 15. Notably, 28 trials (19.7%) scored excellent, indicating inclusivity with respect to eligibility criteria, whereas 34 trials (23.9%) scored poor, indicating more exclusive eligibility criteria.

**Figure 3:**
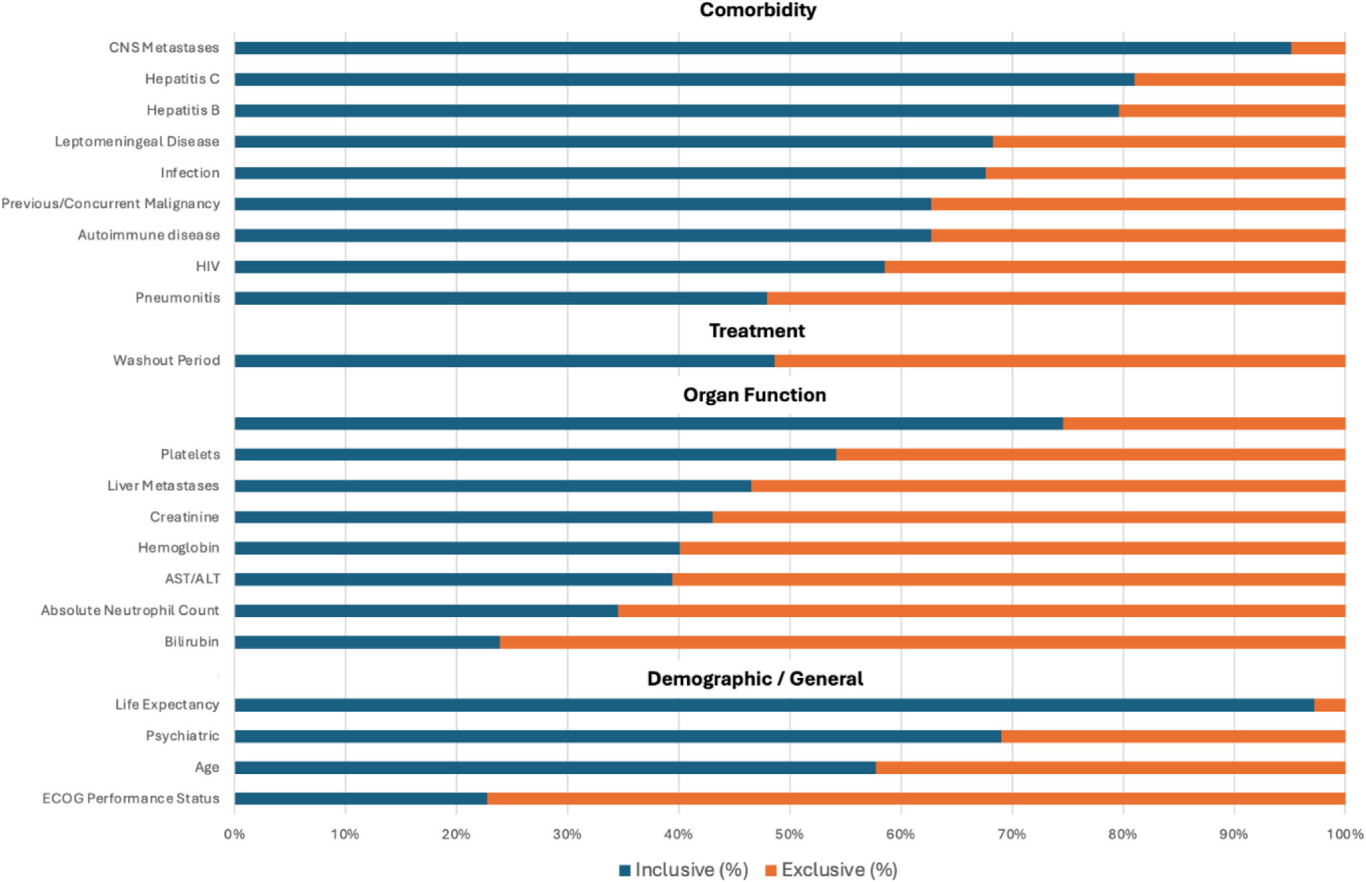
Proportion of inclusive versus exclusive eligibility criteria in advanced NSCLC immunotherapy trials. ALT, alanine aminotransferase; AST, aspartate aminotransferase; CNS, central nervous system; ECOG, Eastern Cooperative Oncology Group; HIV, human immunodeficiency virus.

Figure 3 and Supplementary Table 1 provide the number and percentage of trials classified as either inclusive or exclusive for each of the 22 ICTIS criteria. Among the highly exclusive criteria were ECOG performance status (78.8%) and organ function criteria of bilirubin (76.1%) and absolute neutrophil count (ANC) (65.5%). Despite the high exclusivity with respect to ECOG performance status, studies were generally more inclusive with other demographic criteria. Trials report greater inclusivity for patients with advanced age (97.2%).

Among organ functions, we found cardiac function to be generally inclusive among trials. For this criterion, trials that used a validated clinical classification system, such as the New York Heart Association Functional Classification, and excluded low-risk cardiac events occurring within 3 months were considered inclusive. Notably, 74.6% of trials were inclusive with regard to cardiac criteria. In contrast, organ function on the basis of aspartate aminotransferase (AST) or alanine aminotransferase (ALT) (60.6%), bilirubin (76.1%), and ANC (65.5%) was highly exclusive. In addition, platelets and hemoglobin threshold were also moderately exclusive in 45.8% and 59.5% of trials with requirements for hemoglobin greater than 8 g/dL, platelet counts higher than 75,0000 cells/uL for immunotherapy-only trials, and platelet counts higher than 100,000 cells/uL for all others.

Among comorbidities, central nervous system metastasis was generally inclusive, with 95% of trials allowing patients with asymptomatic or treated brain metastasis to be included. Criteria related to infection, HIV, hepatitis B, and hepatitis C were largely inclusive, with 67.6%, 83.5%, 79.6%, and 81.0% of trials being inclusive, respectively.

### Trends in Eligibility Criteria and ICTIS Scores Over Time

Leptomeningeal criteria became significantly more inclusive over the years from the release of the 2017 ASCO guidelines, recommending inclusion of patients with treated leptomeningeal disease (*p* = 0.018). Only 16.7% of trials used inclusive leptomeningeal disease criteria before 2017 compared with 72.7% from 2018 to 2020 and 68.8% from 2021 to 2023. The other 21 out of 22 criteria were not significantly different across the start year (Supplementary Table 2).

**Figure 4:**
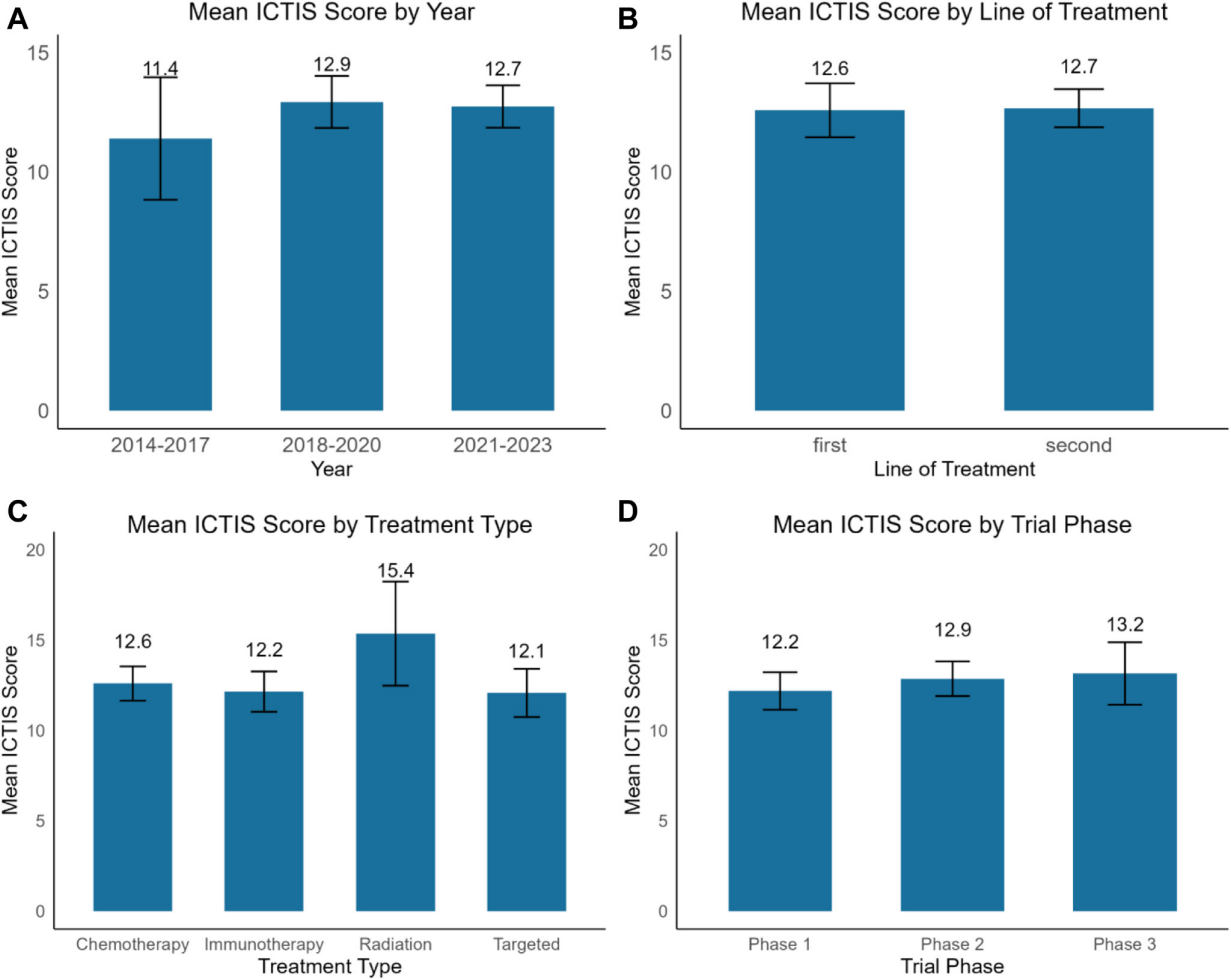
Comparison of mean ICTIS scores across (*A*) time period, (*B*) line of treatment, (*C*) treatment type, and (*D*) trial phase. No significant differences were found across all groups (*t* test or ANOVA, *p* > 0.05). All error bars represent plus or minus 2 SE. ANOVA, analysis of variance; ICTIS, immunotherapy clinical trial inclusivity scale.

The mean ICTIS score increased over time (Fig. 4*A*), with 11.4 for 2014 to 2017, 12.94 for 2018 to 2020, and 12.74 for 2021 to 2023. However, there was no statistically significant difference in scores between the time periods, suggesting that, although there was an apparent improvement in ICTIS scores over time, it did not represent a meaningful improvement in trial inclusivity (*p* = 0.53).

### Exclusive Pneumonitis Criteria in First-Line Trials With No Significant Difference in ICTIS Scores

We evaluated whether there were differences in eligibility criteria by line of treatment. A significantly higher proportion of first-line trials had exclusive pneumonitis criteria, defined as a history of pneumonitis, than second-line trials (χ^2^ = 4.92, *p* = 0.027). There were 63.8% of first-line trials that used exclusive pneumonitis criteria compared with 45.5% of second-line trials. The remaining 21 out of 22 of the criteria were not significantly different across different lines of treatment (Supplementary Table 3). As illustrated in Figure 4*B*, the mean ICTIS scores were similar (*p* = 0.90), with no significant difference between first-line trials (12) and second-line trials (12.7).

### Exclusive Platelet Criteria in Immunotherapy Monotherapy Trials

A significantly higher proportion of immunotherapy-only trials used exclusive platelet criteria compared with trials combining immunotherapy with chemotherapy, targeted therapy, or radiation (χ^2^ = 11.9, *p* = 0.007). Specifically, 69.6% of monotherapy trials had exclusive platelet criteria, compared with immunotherapy trials combined with chemotherapy (39.7%), targeted therapy (39.5%), and radiation (9.1%). The most common threshold was a minimum of 100,000 platelets/μL for inclusion in these monotherapy trials (n = 43), which is considered exclusive in that context. The remaining ICTIS criteria were not significantly different across different types of combination treatments (Supplementary Table 4).

Figure 4*C* illustrates mean ICTIS scores for immunotherapy only (12.15), chemotherapy combination (12.60), targeted therapy combination (12.08), and radiation combination (15.36). The mean ICTIS score was not significantly different between treatment types (*p* = 0.08).

### Exclusive Cardiac Criteria in Early-Phase Clinical Trials

A significantly higher proportion of early-phase trials required restrictive cardiac criteria compared with phase 3 trials (χ^2^=5.10, *p* = 0.024). Specifically, 28.9% of phase 1 or 2 trials had exclusive cardiac disease criteria—often on the basis of vague or unclear exclusions, such as not using a validated clinical classification system or excluding low-risk cardiac events occurring more than 3 months prior, whereas only 7.7% of phase 3 trials had similar exclusions. The remaining 20 out of 22 ICTIS criteria found no significant differences across the trial phases (Supplementary Table 5).

Figure 4*D* displays the mean ICTIS score for phase 1 (12.19), phase 2 (12.87), and phase 3 (13.15) trials. There was no significant difference between the mean ICTIS scores for phases (*p* = 0.51).

### Concordance of ClinicalTrials.gov Data With Protocol-Defined Criteria

A total of 17 out of 142 ICTIS trials had publicly available protocols. An average change of −1.17 points was found when comparing ICTIS scores on the basis of ClinicalTrials.gov data and full protocol data. Scores between the full protocols and the ClinicalTrials.gov protocol did not reveal a significant difference (*p* = 0.208).

Table 3 displays the change in ICTIS score and the change in ICTIS score for specific categories for the 17 clinical trials that had full protocol data.

**Table 3:** Comparison of ICTIS Scores on the Basis of Clinicaltrials.gov Criteria Versus Full Protocol Criteria for the 17 Trials With Full Protocol Data

NCT Identifier	Original ICTIS Score	New ICTIS Score	Demographic and General	Organ Function	Treatment and Washout	Comorbidities
NCT03829501	8	8	0	0	0	0
NCT03953235	11	10	0	0	0	-1
NCT04032704	20	16	0	-4	0	0
NCT04875806	9	9	0	0	0	0
NCT05397171	10	10	0	1	0	-1
NCT03739710	9	12	0	3	0	0
NCT04585815	8	10	2	0	0	0
NCT03847519	8	9	0	1	0	0
NCT04533451	18	18	0	0	0	0
NCT04043195	11	1	0	-7	0	-3
NCT04249362	14	14	0	0	0	0
NCT03164616	10	4	-1	-5	0	0
NCT04294810	8	9	0	1	0	0
NCT05171777	14	7	0	-5	0	-2
NCT04964960	8	9	0	2	0	-1
NCT05502237	9	10	0	1	0	0
NCT02681549	6	6	0	0	0	0

Each trial is identified by its NCT number, with the "Original ICTIS Score" derived from ClinicalTrials.gov and the "New ICTIS Score" recalculated using the full protocol. Changes are broken down into four categories: demographic and general, organ function, treatment and washout, and comorbidities. Whereas most trials reported minimal or no change, a few exhibited notable differences, primarily because of more detailed organ function criteria in the full protocols. Overall, the ICTIS scores were largely concordant between the two sources. However, when discrepancies did occur, they tended to reflect more exclusive criteria in the full protocols, suggesting that ClinicalTrials.gov may underreport certain restrictive eligibility details. Color coding in the table highlights these shifts: red for negative (more exclusive) changes, green for positive (more inclusive) changes, and blue for changes with no net impact on the overall score.

ICTIS, immunotherapy clinical trial inclusivity scale; NCT, National Clinical Trial number.

## Discussion

Currently, fewer than 5% of patients with cancer participate in clinical trials, with an estimated 17% to 21% excluded because of restrictive eligibility criteria.^15^^,^^21^ Harvey et al.^20^ analyzed ASCO’s CancerLinQ database and found that 48% of patients with advanced NSCLC from a sample of 5005 would be excluded on the basis of just three criteria, namely: (1) exclusion of previous or concurrent malignancies, (2) brain metastases, and (3) poor renal function. Similarly, Tang et al.^22^ conducted a population-based cohort study in 2021 in patients with advanced NSCLC (≥ 65 years old) and found that 53.4% of the patients would be excluded because of inadequate organ function and low performance status. However, the authors also found no significant difference in treatment uptake between trial-eligible and ineligible older patients. This finding suggests that real-world cancer treatment often includes both trial-typical and ineligible patients, indicating that trials may not represent the broader patient population.

The goal of this study is to identify common restrictive criteria and evaluate the overall inclusivity of recruiting NSCLC immunotherapy clinical trials. To facilitate evaluation of clinical trial inclusivity, we developed a scoring tool, ICTIS, on the basis of current recommendations from national organizations.

### Overview of Eligibility Criteria Inclusivity

We found that ECOG performance status was the most restrictive across clinical trials. Specifically, 78.8% of clinical trials required ECOG 0 to 1, whereas only 23.2% allowed for a more inclusive performance status without stringent restrictions. In contrast, a 2017 study by Jin et al.,^23^ which analyzed eligibility criteria in investigational new drug applications submitted to the U.S. FDA, found that 63% of trials used ECOG 0 to 1 and 37% used ECOG 0 to 2-plus. This suggests that current NSCLC immunotherapy trials are adopting equally stringent inclusion criteria for performance status compared with earlier trials from 2015.

Our results are comparable to those reported in the study by Jin et al., in which trials had restrictive requirements for eligibility, such as hemoglobin above 8 g/dL, AST or ALT below three times the upper limit of normal (ULN), creatinine clearance above 30 mL/min, and ANC above 1000 cells/μL. We found that these restrictive requirements persist in the current immunotherapy clinical trial landscape, with frequent exclusions on the basis of AST or ALT (60.6%), bilirubin (76.1%), and ANC (65.5%). However, such stringent parameters are often less pertinent to immunotherapy trials, given the different safety profiles of immunotherapy compared with cytotoxic chemotherapies.^10^^,^^11^ For instance, the risk for cytopenia with immunotherapy is less than 5% of immunotherapy patients.^24^ Moreover, organ function criteria for ANC and creatinine clearance frequently exclude African American patients who physiologically have lower ANC and creatinine clearance, thereby disproportionately impacting minority patients.^25^

Despite high exclusion on the basis of ECOG, studies were generally more inclusive with respect to age, asymptomatic or treated central nervous system metastases, hepatitis B, and hepatitis C. Using ICTIS, we found that 97.2% of clinical trials used inclusive age criteria, which is nearly identical to the 97.9% reported by Jin et al. in 2015, with an upper age limit of 85 years or older. In addition, 95.1% of NSCLC immunotherapy trials included patients with treated brain metastases. This is a significant increase from the 47.1% noted in the study by Jin et al. The inclusion of patients with hepatitis B and C also saw substantial increases, with 79.6% and 81.0% of trials being inclusive, compared with only 30% in 2015. Overall, NSCLC immunotherapy trials have become more inclusive with regard to brain metastases and hepatitis compared with earlier trials.

### ICTIS Scores and Subgroup Analyses on the Inclusivity of NSCLC Immunotherapy Trials

The overall mean ICTIS score across all 142 trials was 12.7 with a SD of four, a median of 12, and an IQR of 10 to 15. Most NSCLC immunotherapy clinical trials still fall short in terms of inclusivity. In fact, only 28 trials (19.7%) scored excellent, indicating inclusive eligibility criteria.

When we evaluated the eligibility criteria of NSCLC immunotherapy trials over time, treated leptomeningeal disease was the only criterion that exhibited significant improvement since the release of the 2017 ASCO guidelines that called for the inclusion of these patients. The overall inclusivity of NSCLC immunotherapy trials has not improved, reflected in the similar scores over time.

By comparing trials by line of treatment, we found that trials evaluating first-line treatment were more exclusive to patients with a history of pneumonitis compared with trials evaluating second-line treatment. Although some studies specifically state that patients with previous noninfectious pneumonitis are excluded, many use pneumonitis as a vague exclusion criterion. Given that many patients with NSCLC have consolidative lung masses mimicking pneumonitis or have a history of pneumonitis from infectious processes, radiation, or as a nonimmunologic reaction to previous treatments, trials should specify immune-related pneumonitis as an exclusion. Indeed, numerous recommendations have been made for second-line trials to include these patients to better reflect the real-world patient population.^10^ Reflecting these recommendations, we saw that a higher proportion of second-line trials have adopted a more inclusive pneumonitis criterion compared with first-line trials. However, second-line trials were equally restrictive as first-line trials with regard to organ function. This was a surprising finding because patients on second-line treatments often have abnormal laboratory parameters for organ function owing to the effects of previous lines of treatment, comorbidities, and underlying progressive malignancy.^23^

Given that agents such as chemotherapy or tyrosine kinase inhibitors used in clinical trials involving immunotherapy in NSCLC can increase toxicity risks, stricter eligibility criteria may be necessary for combination regimens. To evaluate this, we compared ICTIS criteria between trials investigating immunotherapy-only versus in combination with other therapies. Surprisingly, we found that immunotherapy-only trials were significantly more restrictive for certain criteria unrelated to immunotherapy toxicity, such as requiring a threshold of 100,000 platelets/μL, which is typically used in combination treatments involving chemotherapy, targeted therapy, or radiation.^24^ Given that immunotherapy is not associated with significant hematologic toxicity, LUNGevity recommends a platelet threshold of 75,000 platelets/μL.^10^ In addition, the other 21 out of 22 ICTIS criteria, including other hematologic parameters such as ANC and hemoglobin, were equally restrictive in immunotherapy-only trials compared with combination therapies. Although the risk for cytopenia occurs in less than 5% of immunotherapy patients, the uniformity of hemoglobin parameters across all trial types suggests limited efforts to tailor eligibility criteria on the basis of different risk profiles of immunotherapy-only versus in combination with other treatments.

Furthermore, we also compared trials by phase as we expected phase 3 trials to have established the general safety of the investigational agent, and therefore, should have less stringent eligibility criteria to accrue the most patients.^20^^,^^23^ Our findings revealed that cardiac restrictions became less stringent in phase 3 trials. Whereas 28.9% of early-phase trials enforced restrictive cardiac exclusions, often on the basis of vague or nonstandardized criteria, such as excluding patients without validated clinical classifications or those with low-risk cardiac events more than 3 months prior, only 7.7% of phase 3 trials maintained such restrictive cardiac criteria. However, the remaining 20 out of 22 ICTIS criteria and mean ICTIS scores revealed no significant differences across trial phases, indicating that the rest of the eligibility criteria were treated uniformly across all phases of trials. Expanding eligibility criteria to be more inclusive, especially in later phases, could improve trial accessibility, ensure a more representative patient population, and promote the generalizability of phase 3 results.

### ClinicalTrials.gov as a Valid Data Source for ICTIS Analysis

ICTIS scores did not see a significant difference when applied between full protocols and ClinicalTrials.gov criteria (n = 17, *p* = 0.208). Most changes in the ICTIS score were because of differences in the specificity of the organ function criteria in the full protocol. Many trials on ClinicalTrials.gov do not list specific organ function parameters that were found in the full protocol. This limits trial accessibility as patients are not able to fully determine which trials they are eligible for on the basis of listed criteria. Investigators should list full organ function criteria on ClinicalTrials.gov. Demographic and general criteria and comorbidity saw little change, and treatment criteria saw no change (Table 3).

A few trials had a significant change in the ICTIS score. NCT04043195 changed from a score of 11 to 1 after reviewing the full protocol, and its categorization went from good to poor. The differences were primarily from organ function (exclusive platelets, ANC, hemoglobin, creatinine, AST or ALT, and bilirubin) and comorbidity (HIV, hepatitis) exclusion criteria that weren’t listed on ClinicalTrials.gov. This study did not properly reflect its protocol on ClinicalTrials.gov. NCT03164616 saw a change from 10 to 4, and NCT05171777 changed from 14 to 7, both trials primarily experiencing differences in organ function criteria.

Overall, most trials saw little to no change in ICTIS score, and differences in criteria were trivial (Fig. 5). ICTIS’ score reflects information relevant to patients looking to enroll in clinical trials, as full protocols are not easily accessible. ClinicalTrials.gov remains a widely accepted and standard source of eligibility criteria, particularly for ongoing and not-yet-recruiting studies. Since the FDA Amendments Act, interventional trials are mandated to report protocol-specified eligibility criteria at the time of registration. Unlike other aspects of trial designs, such as end points or sample size, eligibility criteria are generally stable and not expected to undergo major revision after registration. The purpose of our analysis is to capture high-level patterns in trial inclusivity rather than protocol-level granularity. As such, ClinicalTrials.gov offers a valid and representative data source for this analysis.

### Limited Adoption of National Guidelines to Promote Inclusivity in Immunotherapy Clinical Trials

Several factors likely contribute to the limited adoption of eligibility guidelines for immunotherapy trials, including concerns for unpredictable toxicities for patients and interference with overall study results. Investigators may perceive broadening eligibility criteria as increasing heterogeneity, potentially introducing bias that could complicate data interpretation or increase the risk of adverse events. These concerns may explain why adoption of these recommendations has been especially limited in early-phase trials, in which safety signals and feasibility are critical. In addition, practical challenges with recruiting patients with comorbidities or limited performance status, along with the need for stringent safety monitoring in patients deemed to have borderline organ function, may discourage adoption of these recommendations.

Regulatory agencies and sponsors emphasize trial success and expedited approval pathways, creating pressures that may discourage sponsors from adopting broader eligibility criteria because of concerns about longer recruitment timelines and increased complexity in trial protocol. The reliance on historically established eligibility criteria, such as ECOG performance status and organ function parameters, also reflects a reluctance to reform, particularly given the high-stakes nature of clinical trials involving novel immunotherapy agents.

Our findings indicate that dissemination of guidelines from national organizations has not necessarily translated into widespread uptake and implementation in the clinical trial landscape. Bridging this gap will require greater collaboration between national organizations, regulatory agencies, trial sponsors, and investigators. Further research is needed to explore why national guidelines have not resulted in broader inclusivity in clinical trial eligibility. A mixed-methods approach that investigates barriers to implementation, including stakeholder awareness, perspectives, and perceived risks, will be beneficial in offering actionable insights. Within this approach, ICTIS can serve as a quantitative tool to objectively evaluate inclusivity, track changes, and benchmark inclusivity across clinical trials. Furthermore, integrating ICTIS scores into regulatory and funding mechanisms may encourage sponsors to design more inclusive trials without compromising scientific rigor.

Although increasing clinical trial inclusivity could broaden access and improve the real-world applicability of findings, we recognize that current eligibility criteria serve important functions. These criteria are not only designed to protect vulnerable patients from potential harm, but also to help ensure that trials can reliably reveal the survival benefits of investigational therapies.^9^ In some cases, including patients with advanced age, poor performance status, or comorbidities, may introduce confounding factors that obscure treatment effects, necessitate larger sample sizes, and increase trial costs without necessarily improving outcomes. Furthermore, we acknowledge that drug approvals are typically broad and not limited by trial inclusion criteria, and that real-world data (RWD) can help address gaps in evidence for underrepresented groups. Ideally, trial design would balance inclusivity with feasibility and scientific rigor to maximize both patient safety and the ability to generate actionable evidence.

### Study Limitations and Future Directions

Although the ICTIS score provides a structured approach to evaluating the rigor of cancer trial eligibility criteria, it is not without limitations. The score focuses primarily on the restrictiveness of inclusion and exclusion criteria, which may not capture all dimensions of trial quality or generalizability. In addition, the score has not yet been validated across diverse cancer types or trial phases, which may limit its broader applicability.

Future research should aim to refine and validate the ICTIS score across a wider range of oncology trials and explore its correlation with trial outcomes and postapproval effectiveness. Moreover, although clinical trial criteria are necessarily rigid to ensure internal validity, the real-world application of approved therapies often extends to a broader and more diverse patient population. Therefore, integrating RWD can help address the gaps between trial populations and actual clinical practice. RWD can serve as a complementary tool to assess the external validity of trials and guide more inclusive trial designs in the future.

In conclusion, our results indicate that despite national guidelines released in the past 6 years aimed at improving inclusivity, immunotherapy trials have made limited progress in broadening eligibility criteria. Among the top three most exclusive criteria were ECOG performance status, organ function criteria of bilirubin, and ANC. Most trials, regardless of treatment type, line, or phase, continue to use similarly restrictive eligibility criteria. Evidence-based frameworks such as the ICTIS scoring system provide a structured approach to evaluating clinical trial inclusivity and support the design of clinical trials that better reflect real-world patient populations while maintaining scientific rigor.

## CRediT Authorship Contribution Statement

**Kira Nguyen:** Conceptualization, Methodology, Investigation, Data curation, Analysis, Writing- Original draft, Revised version, Review and editing.

**Ashley Wei:** Methodology, Investigation, Data curation, Analysis, Writing- Original draft, Revised version, Review and editing.

**Srinivas Govindan:** Validation, Writing – review and editing.

**Eziafa Oduah:** Methodology, Investigation, Writing – review and editing.

**Nagashree Seetharamu:** Conceptualization, Methodology, Supervision, Writing – review and editing.

**Wint Yan Aung:** Conceptualization, Methodology, Investigation, Writing - original draft, Writing – review and editing.

## Disclosure

The authors declare no conflict of interest.
