# Supplementary material for: ICTIS: A Novel Scoring System to Assess the Inclusivity of Advanced NSCLC Immunotherapy Trials
Source: JTO Clin Res Rep. 2025 Jul 12;6(11):100878. doi: 10.1016/j.jtocrr.2025.100878 (PMC12614445; doi:10.1016/j.jtocrr.2025.100878)
Supplement: Supplementary Tables 1-5 [file mmc1.docx]

**Supplemental Tables**

| **Table S1.** Frequency of inclusive and exclusive eligibility criteria in recruiting, and not-yet-recruiting immunotherapy clinical trials for advanced NSCLC accessed through ClinicalTrials.gov (N = 142) | | |
| --- | --- | --- |
| **Demographic / General** | **Inclusive n (%)** | **Exclusive n (%)** |
| ECOG Performance Status | 33 (23.2) | 109 (78.8) |
| Psychiatric | 98 (69.0) | 44 (31.0) |
| Age | 82 (57.7) | 60 (42.3) |
| Life Expectancy | 138 (97.2) | 4 (2.8) |
| **Organ Function** |  |  |
| Platelets | 77 (54.2) | 65 (45.8) |
| Hemoglobin | 57 (40.1) | 85 (59.9) |
| AST/ALT | 56 (39.4) | 86 (60.6) |
| Liver Metastases | 66 (46.5) | 76 (53.5) |
| Creatinine | 61 (43.0) | 81 (57.0) |
| Bilirubin | 34 (23.9) | 108 (76.1) |
| Absolute Neutrophil Count | 49 (34.5) | 93 (65.5) |
| Cardiac | 106 (74.6) | 36 (25.4) |
| **Treatment** |  |  |
| Washout Period | 69 (48.6) | 73 (51.4) |
| **Comorbidity** |  |  |
| Pneumonitis | 68 (47.9) | 74 (52.1) |
| Previous/Concurrent Malignancy | 89 (62.7) | 53 (37.3) |
| CNS Metastases | 135 (95.1) | 7 (4.9) |
| Leptomeningeal Disease | 97 (68.3) | 45 (31.7) |
| Infection | 96 (67.6) | 46 (32.4) |
| HIV | 83 (58.5) | 59 (41.5) |
| Hepatitis B | 113 (79.6) | 29 (20.4) |
| Hepatitis C | 115 (81.0) | 27 (19.0) |
| Autoimmune disease | 89 (62.7) | 53 (37.3) |

| **Table S2.** Comparison of criteria based on study period | | | | | |
| --- | --- | --- | --- | --- | --- |
| **General Criteria** | **Pre-2017**  **N= 6** | **2017-2020**  **N = 66** | **2021**  **N = 70** | **Chi- Square** | **P-Value** |
| ECOG Inclusive | 1 | 12 | 20 |  |  |
| ECOG Exclusive | 5 | 54 | 50 |  |  |
|  |  |  |  | 2.207 | 0.332 |
| Psychiatric Inclusive | 4 | 48 | 46 |  |  |
| Psychiatric Exclusive | 2 | 18 | 24 |  |  |
|  |  |  |  | 0.797 | 0.671 |
| Life Expectancy Inclusive | 2 | 39 | 41 |  |  |
| Life Expectancy Exclusive | 4 | 27 | 29 |  |  |
|  |  |  |  | 1.534 | 0.464 |
| Age Inclusive | 5 | 65 | 68 |  |  |
| Age Exclusive | 1 | 1 | 2 |  |  |
|  |  |  |  | 4.613 | 0.100 |
| **Organ Function Criteria** |  |  |  |  |  |
| Platelets Inclusive | 3 | 38 | 36 |  |  |
| Platelets Exclusive | 3 | 28 | 34 |  |  |
|  |  |  |  | 0.562 | 0.755 |
| Hemoglobin Inclusive | 1 | 29 | 27 |  |  |
| Hemoglobin Exclusive | 5 | 37 | 43 |  |  |
|  |  |  |  | 1.844 | 0.398 |
| AST/ALT Inclusive | 2 | 28 | 26 |  |  |
| AST/ALT Exclusive | 4 | 38 | 44 |  |  |
|  |  |  |  | 0.494 | 0.781 |
| Liver Metastases Inclusive | 2 | 33 | 31 |  |  |
| Liver Metastases Exclusive | 4 | 33 | 39 |  |  |
|  |  |  |  | 0.881 | 0.644 |
| Creatinine Inclusive | 2 | 29 | 30 |  |  |
| Creatinine Exclusive | 4 | 37 | 40 |  |  |
|  |  |  |  | 0.253 | 0.881 |
| Bilirubin Inclusive | 0 | 15 | 19 |  |  |
| Bilirubin Exclusive | 6 | 51 | 51 |  |  |
|  |  |  |  | 2.336 | 0.311 |
| ANC Inclusive | 1 | 24 | 24 |  |  |
| ANC Exclusive | 5 | 42 | 46 |  |  |
|  |  |  |  | 0.947 | 0.623 |
| Cardiac Inclusive | 6 | 49 | 51 |  |  |
| Cardiac Exclusive | 0 | 17 | 19 |  |  |
|  |  |  |  | 2.162 | 0.339 |
| **Treatment Criteria** |  |  |  |  |  |
| Washout Inclusive | 3 | 32 | 34 |  |  |
| Washout Exclusive | 3 | 34 | 36 |  |  |
|  |  |  |  | 0.005 | 0.997 |
| **Comorbidities** |  |  |  |  |  |
| Pneumonitis Inclusive | 3 | 34 | 31 |  |  |
| Pneumonitis Exclusive | 3 | 32 | 39 |  |  |
|  |  |  |  | 0.723 | 0.697 |
| Malignancy Inclusive | 5 | 43 | 41 |  |  |
| Malignancy Exclusive | 1 | 23 | 29 |  |  |
|  |  |  |  | 1.772 | 0.412 |
| CNS Inclusive | 5 | 63 | 67 |  |  |
| CNC Exclusive | 1 | 3 | 3 |  |  |
|  |  |  |  | 1.846 | 0.397 |
| Leptomeningeal Disease Inclusive | 1 | 48 | 48 |  |  |
| Leptomeningeal Disease Exclusive | 5 | 18 | 22 |  |  |
|  |  |  |  | **7.989** | **0.018** |
| Infection Inclusive | 3 | 41 | 52 |  |  |
| Infection Exclusive | 3 | 25 | 18 |  |  |
|  |  |  |  | 3.182 | 0.204 |
| HIV Inclusive | 1 | 38 | 44 |  |  |
| HIV Exclusive | 5 | 28 | 26 |  |  |
|  |  |  |  | 4.894 | 0.087 |
| Hepatitis B Inclusive | 4 | 55 | 54 |  |  |
| Hepatitis B Exclusive | 2 | 11 | 16 |  |  |
|  |  |  |  | 1.444 | 0.486 |
| Hepatitis C Inclusive | 3 | 57 | 55 |  |  |
| Hepatitis C Exclusive | 3 | 9 | 15 |  |  |
|  |  |  |  | 5.246 | 0.073 |

| **Table S3**. Comparison of criteria based on first line or second line therapy | | | | |
| --- | --- | --- | --- | --- |
| **General Criteria** | **First Line**  **N = 58** | **Second Line**  **N = 101** | **Chi-Square** | **P-Value** |
| ECOG Inclusive | 16 | 21 |  |  |
| ECOG Exclusive | 42 | 80 |  |  |
|  |  |  | 0.953 | 0.329 |
| Psychiatric Inclusive | 37 | 41 |  |  |
| Psychiatric Exclusive | 21 | 30 |  |  |
|  |  |  | 0.817 | 0.366 |
| Life Expectancy Inclusive | 30 | 63 |  |  |
| Life Expectancy Exclusive | 28 | 38 |  |  |
|  |  |  | 1.749 | 0.186 |
| Age Inclusive | 57 | 97 |  |  |
| Age Exclusive | 1 | 4 |  |  |
|  |  |  | 0.735 | 0.391 |
| **Organ Function Criteria** |  |  |  |  |
| Platelets Inclusive | 33 | 53 |  |  |
| Platelets Exclusive | 25 | 48 |  |  |
|  |  |  | 0.291 | 0.589 |
| Hemoglobin Inclusive | 25 | 39 |  |  |
| Hemoglobin Exclusive | 33 | 62 |  |  |
|  |  |  | 0.310 | 0.578 |
| AST/ALT Inclusive | 27 | 65 |  |  |
| AST/ALT Exclusive | 31 | 36 |  |  |
|  |  |  | 1.838 | 0.175 |
| Liver Metastases Inclusive | 31 | 43 |  |  |
| Liver Metastases Exclusive | 27 | 58 |  |  |
|  |  |  | 1.752 | 0.186 |
| Creatinine Inclusive | 25 | 41 |  |  |
| Creatinine Exclusive | 33 | 60 |  |  |
|  |  |  | 0.231 | 0.631 |
| Bilirubin Inclusive | 16 | 22 |  |  |
| Bilirubin Exclusive | 42 | 79 |  |  |
|  |  |  | 0.682 | 0.409 |
| ANC Inclusive | 20 | 35 |  |  |
| ANC Exclusive | 38 | 66 |  |  |
|  |  |  | 0.001 | 0.975 |
| Cardiac Inclusive | 40 | 75 |  |  |
| Cardiac Exclusive | 18 | 26 |  |  |
|  |  |  | 0.998 | 0.318 |
| **Treatment Criteria** |  |  |  |  |
| Washout Inclusive | 28 | 49 |  |  |
| Washout Exclusive | 30 | 52 |  |  |
|  |  |  | 0.003 | 0.960 |
| **Comorbidities** |  |  |  |  |
| Pneumonitis Inclusive | 21 | 55 |  |  |
| Pneumonitis Exclusive | 37 | 46 |  |  |
|  |  |  | **4.917** | **0.027** |
| Malignancy Inclusive | 36 | 63 |  |  |
| Malignancy Exclusive | 22 | 38 |  |  |
|  |  |  | 0.013 | 0.909 |
| CNS Inclusive | 55 | 95 |  |  |
| CNC Exclusive | 3 | 6 |  |  |
|  |  |  | 0.228 | 0.633 |
| Leptomeningeal Disease Inclusive | 34 | 70 |  |  |
| Leptomeningeal Disease Exclusive | 24 | 31 |  |  |
|  |  |  | 2.562 | 0.109 |
| Infection Inclusive | 38 | 70 |  |  |
| Infection Exclusive | 20 | 31 |  |  |
|  |  |  | 0.249 | 0.618 |
| HIV Inclusive | 35 | 57 |  |  |
| HIV Exclusive | 23 | 44 |  |  |
|  |  |  | 0.255 | 0.614 |
| Hepatitis B Inclusive | 44 | 83 |  |  |
| Hepatitis B Exclusive | 14 | 18 |  |  |
|  |  |  | 0.913 | 0.339 |
| Hepatitis C Inclusive | 48 | 81 |  |  |
| Hepatitis C Exclusive | 10 | 20 |  |  |
|  |  |  | 0.159 | 0.690 |

| **Table S4.** Comparison of criteria based on treatment type | | | | | | |
| --- | --- | --- | --- | --- | --- | --- |
| **General Criteria** | **Immunotherapy**  **N = 46** | **Chemotherapy**  **N = 58** | **Radiation**  **N = 11** | **Targeted Therapy**  **N = 38** | **Chi - Square** | **P-Value** |
| ECOG Inclusive | 6 | 12 | 7 | 5 |  |  |
| ECOG Exclusive | 40 | 46 | 4 | 33 |  |  |
|  |  |  |  |  | 5.057 | 0.168 |
| Psychiatric Inclusive | 33 | 43 | 6 | 25 |  |  |
| Psychiatric Exclusive | 13 | 15 | 5 | 13 |  |  |
|  |  |  |  |  | 1.057 | 0.788 |
| Life Expectancy Inclusive | 31 | 29 | 5 | 23 |  |  |
| Life Expectancy Exclusive | 15 | 29 | 6 | 15 |  |  |
|  |  |  |  |  | 3.300 | 0.348 |
| Age Inclusive | 46 | 54 | 11 | 38 |  |  |
| Age Exclusive | 0 | 4 | 0 | 0 |  |  |
|  |  |  |  |  | 5.961 | 0.114 |
| **Organ Function Criteria** |  |  |  |  |  |  |
| Platelets Inclusive | 14 | 35 | 10 | 23 |  |  |
| Platelets Exclusive | 32 | 23 | 1 | 15 |  |  |
|  |  |  |  |  | 11.972 | 0.007 |
| Hemoglobin Inclusive | 16 | 25 | 5 | 12 |  |  |
| Hemoglobin Exclusive | 30 | 33 | 6 | 26 |  |  |
|  |  |  |  |  | 1.921 | 0.589 |
| AST/ALT Inclusive | 16 | 22 | 5 | 15 |  |  |
| AST/ALT Exclusive | 30 | 36 | 6 | 23 |  |  |
|  |  |  |  |  | 0.472 | 0.925 |
| Liver Metastases Inclusive | 20 | 21 | 10 | 17 |  |  |
| Liver Metastases Exclusive | 26 | 37 | 1 | 21 |  |  |
|  |  |  |  |  | 2.673 | 0.445 |
| Creatinine Inclusive | 17 | 23 | 6 | 16 |  |  |
| Creatinine Exclusive | 29 | 35 | 5 | 22 |  |  |
|  |  |  |  |  | 0.946 | 0.814 |
| Bilirubin Inclusive | 10 | 12 | 4 | 8 |  |  |
| Bilirubin Exclusive | 36 | 46 | 7 | 30 |  |  |
|  |  |  |  |  | 0.634 | 0.889 |
| ANC Inclusive | 16 | 18 | 6 | 9 |  |  |
| ANC Exclusive | 30 | 40 | 5 | 29 |  |  |
|  |  |  |  |  | 2.281 | 0.516 |
| Cardiac Inclusive | 32 | 44 | 9 | 30 |  |  |
| Cardiac Exclusive | 14 | 14 | 2 | 8 |  |  |
|  |  |  |  |  | 1.044 | 0.791 |
| **Treatment Criteria** |  |  |  |  |  |  |
| Washout Inclusive | 20 | 33 | 8 | 13 |  |  |
| Washout Exclusive | 26 | 25 | 3 | 25 |  |  |
|  |  |  |  |  | 5.229 | 0.156 |
| **Comorbidities** |  |  |  |  |  |  |
| Pneumonitis Inclusive | 22 | 29 | 11 | 13 |  |  |
| Pneumonitis Exclusive | 24 | 29 | 0 | 25 |  |  |
|  |  |  |  |  | 2.952 | 0.399 |
| Malignancy Inclusive | 25 | 35 | 10 | 23 |  |  |
| Malignancy Exclusive | 21 | 23 | 1 | 15 |  |  |
|  |  |  |  |  | 1.574 | 0.665 |
| CNS Inclusive | 45 | 54 | 10 | 37 |  |  |
| CNC Exclusive | 1 | 4 | 1 | 1 |  |  |
|  |  |  |  |  | 1.652 | 0.648 |
| Leptomeningeal Disease Inclusive | 33 | 41 | 6 | 24 |  |  |
| Leptomeningeal Disease Exclusive | 13 | 17 | 5 | 14 |  |  |
|  |  |  |  |  | 0.868 | 0.833 |
| Infection Inclusive | 31 | 39 | 7 | 24 |  |  |
| Infection Exclusive | 15 | 19 | 4 | 14 |  |  |
|  |  |  |  |  | 0.348 | 0.951 |
| HIV Inclusive | 24 | 34 | 7 | 24 |  |  |
| HIV Exclusive | 22 | 24 | 4 | 14 |  |  |
|  |  |  |  |  | 1.094 | 0.779 |
| Hepatitis B Inclusive | 36 | 46 | 9 | 29 |  |  |
| Hepatitis B Exclusive | 10 | 12 | 2 | 9 |  |  |
|  |  |  |  |  | 0.300 | 0.960 |
| Hepatitis C Inclusive | 37 | 47 | 9 | 30 |  |  |
| Hepatitis C Exclusive | 9 | 11 | 2 | 8 |  |  |
|  |  |  |  |  | 0.112 | 0.990 |

| **Table S5.** Comparison of criteria based of phases of the trials | | | | |
| --- | --- | --- | --- | --- |
| **General Criteria** | **Phase 1 and 2 Trials**  **N = 121** | **Phase 3 Trials**  **N = 26** | **Chi-Square** | **P-Value** |
| ECOG Inclusive | 29 | 5 |  |  |
| ECOG Exclusive | 92 | 21 |  |  |
|  |  |  | 0.270 | 0.603 |
| Psychiatric Inclusive | 79 | 24 |  |  |
| Psychiatric Exclusive | 42 | 2 |  |  |
|  |  |  | 7.382 | 0.007 |
| Life Expectancy Inclusive | 71 | 14 |  |  |
| Life Expectancy Exclusive | 50 | 12 |  |  |
|  |  |  | 0.205 | 0.651 |
| Age Inclusive | 117 | 26 |  |  |
| Age Exclusive | 4 | 0 |  |  |
|  |  |  | 0.859 | 0.354 |
| **Organ Function Criteria** |  |  |  |  |
| Platelets Inclusive | 66 | 14 |  |  |
| Platelets Exclusive | 55 | 12 |  |  |
|  |  |  | 0.007 | 0.936 |
| Hemoglobin Inclusive | 47 | 11 |  |  |
| Hemoglobin Exclusive | 74 | 15 |  |  |
|  |  |  | 0.136 | 0.713 |
| AST/ALT Inclusive | 46 | 10 |  |  |
| AST/ALT Exclusive | 75 | 16 |  |  |
|  |  |  | 0.113 | 0.737 |
| Liver Metastases Inclusive | 55 | 11 |  |  |
| Liver Metastases Exclusive | 66 | 15 |  |  |
|  |  |  | 0.233 | 0.629 |
| Creatinine Inclusive | 51 | 10 |  |  |
| Creatinine Exclusive | 70 | 16 |  |  |
|  |  |  | 0.247 | 0.619 |
| Bilirubin Inclusive | 25 | 9 |  |  |
| Bilirubin Exclusive | 96 | 17 |  |  |
|  |  |  | 2.342 | 0.126 |
| ANC Inclusive | 40 | 9 |  |  |
| ANC Exclusive | 81 | 17 |  |  |
|  |  |  | 0.113 | 0.737 |
| Cardiac Inclusive | 86 | 24 |  |  |
| Cardiac Exclusive | 35 | 2 |  |  |
|  |  |  | 5.101 | 0.024 |
| **Treatment Criteria** |  |  |  |  |
| Washout Inclusive | 54 | 16 |  |  |
| Washout Exclusive | 67 | 10 |  |  |
|  |  |  | 2.506 | 0.113 |
| **Comorbidities** |  |  |  |  |
| Pneumonitis Inclusive | 59 | 11 |  |  |
| Pneumonitis Exclusive | 62 | 15 |  |  |
|  |  |  | 0.361 | 0.548 |
| Malignancy Inclusive | 78 | 12 |  |  |
| Malignancy Exclusive | 43 | 14 |  |  |
|  |  |  | 3.199 | 0.074 |
| CNS Inclusive | 114 | 26 |  |  |
| CNC Exclusive | 7 | 0 |  |  |
|  |  |  | 1.537 | 0.215 |
| Leptomeningeal Disease Inclusive | 82 | 20 |  |  |
| Leptomeningeal Disease Exclusive | 39 | 6 |  |  |
|  |  |  | 0.907 | 0.341 |
| Infection Inclusive | 79 | 18 |  |  |
| Infection Exclusive | 42 | 8 |  |  |
|  |  |  | 0.328 | 0.567 |
| HIV Inclusive | 68 | 18 |  |  |
| HIV Exclusive | 53 | 8 |  |  |
|  |  |  | 1.497 | 0.221 |
| Hepatitis B Inclusive | 98 | 19 |  |  |
| Hepatitis B Exclusive | 23 | 7 |  |  |
|  |  |  | 0.825 | 0.364 |
| Hepatitis C Inclusive | 100 | 20 |  |  |
| Hepatitis C Exclusive | 21 | 6 |  |  |
|  |  |  | 0.495 | 0.482 |
